# Methodological challenges in using human umbilical artery as a model for in vitro studies

**DOI:** 10.1113/EP091374

**Published:** 2023-10-14

**Authors:** Milica Gajić Bojić, Đorđe Đukanović, Sonja Marinković, Sanja Jovičić, Miloš P. Stojiljković, Dragan M. Djuric, Ranko Škrbić

**Affiliations:** ^1^ Centre for Biomedical Research, Faculty of Medicine University of Banja Luka Banja Luka The Republic of Srpska Bosnia and Herzegovina; ^2^ Department of Paediatrics University Clinical Centre of the Republic of Srpska Banja Luka The Republic of Srpska Bosnia and Herzegovina; ^3^ Department of Histology and Embryology, Faculty of Medicine University of Banja Luka Banja Luka The Republic of Srpska Bosnia and Herzegovina; ^4^ Department of Pharmacology, Toxicology and Clinical Pharmacology, Faculty of Medicine University of Banja Luka Banja Luka The Republic of Srpska Bosnia and Herzegovina; ^5^ Faculty of Medicine, Institute of Medical Physiology ‘Richard Burian’ University of Belgrade Belgrade Serbia

**Keywords:** experimental protocols, organ bath system, umbilical cord, vasoactivity

## Abstract

Human umbilical artery (HUA) preparations are of particular importance for in vitro studies on isolated blood vessels because their sampling is not risky for the patient, and they can provide the closest possible impression of changes related to the uteroplacental circulation during pre‐eclampsia. Using organ bath techniques, useful experimental protocols are provided for measuring some pathophysiological phenomena in the vascular responses of HUAs. Several vasoconstrictors (serotonin, prostaglandin F and phenylephrine) and vasodilators (acetylcholine and minoxidil) were seleted for determination of their vasoactivity in HUAs. The role of L‐type voltage‐operated calcium channels and different types of potassium channels (K_ATP_, BK_Ca_ and K_V_) were assessed, as was the impact of homocysteine. Serotonin was confirmed to be the most potent vasoconstrictor, while acetylcholine and phenylephrine caused variability in the relaxation and contraction response of HUA, respectively. The observed increase in serotonin‐induced contraction and a decrease in minoxidil‐induced relaxation in the presence of homocysteine suggested its procontractile effect on HUA preparations. Using selective blockers, it was determined that K_ATP_ and K_V_ channels participate in the minoxidil‐induced relaxation, while L‐type voltage‐dependent Ca^2+^ channels play an important role in the serotonin‐induced contraction. The presented protocols reveal some of the methodological challenges related to HUA preparations and indicate potential outcomes in interpreting the vascular effects of the investigated substances, both in physiological conditions and in the homocysteine‐induced pre‐eclampsia model.

## Abstract (highlights)

- **What is the central question of this study?**
  What are the biggest challenges in performing in vitro studies on isolated human umbilical arteries?
- **What is the main finding and its importance?**
  The protocols presented in this study indicate some potential outcomes important for interpretation of the vascular responsivities of human umbilical arteries and could be useful for planning future in vitro studies with human umbilical arteries.

## 1 INTRODUCTION

An organ bath system is a very useful methodology for studying the effects of various compounds on isolated blood vessels. By determining the potency and efficacy of vasoactive compounds, it is possible to examine the mechanisms of vasoconstriction or vasodilatation (Jin et al., 2018). Although numerous studies have been performed on isolated animal blood vessels, this cannot adequately mimic the pathophysiological phenomena present in the human body, such as the vascular reactivity of the uteroplacental and fetoplacental circulation.

Preparations obtained from umbilical cords serve as a tool for important research fields, such as models for in vitro study of cardiovascular diseases, including pre‐eclampsia (Medina Leyte et al., 2020). Moreover, in vitro searches for new substances with relaxant properties are based on the use of isolated umbilical arteries and veins (Đukanović et al., 2022). In addition to being an excellent model for testing the vasoactivity of substances, the advantage of the umbilical blood vessels is based primarily on their good availability and harmlessness for the patient, because the umbilical cord is treated as biological waste (Stefos et al., 2003). Therefore, human umbilical artery (HUA) preparations provide a wide range of possibilities for pharmacological investigations of vascular activity, which do not endanger either mother or newborn (Evaristo et al., 2020). Bearing in mind that clinical studies on pregnant women are sporadic and that the interpretation of results from animal models is insufficiently reliable, experiments on HUAs have a special place in pre‐eclampsia studies (Altura et al., 1972; Medina Leyte et al., 2020; Pereira‐de‐Morais et al., 2023).

Given that the umbilical cord is not innervated, the blood flow and resistance in the HUA is regulated exclusively by autacoids from the circulation, such as serotonin, histamine, bradykinin, angiotensin and oxytocin, in addition to various eicosanoids (Bertrand et al., 1993; McGrath et al., 1988; Santos‐Silva et al., 2008). It is well established that the mechanical properties and reactivity of the umbilical arteries and veins are altered in pre‐eclampsia (Altura et al., 1972; Bertrand et al., 1993). Homocysteine‐induced endothelial damage was previously described as a pre‐eclampsia model on isolated HUAs, because it has long been established that hyperhomocysteinaemia is a major and independent risk factor for vascular disease (Okatani et al., 2000). Oxidative stress is a possible mechanism for homocysteine‐induced endothelial dysfunction, as it was found that vascular tension in the HUA is potentiated by homocysteine, most probably by suppression of the endothelial synthesis of NO with peroxides (Upchurch et al., 1997).

To perform in vitro studies on isolated HUAs, it is extremely important to establish optimal experimental conditions, in addition to adequate experimental protocols that will enable the interpretation of changes found in pre‐eclampsia. The protocols presented in this study aim to indicate potential outcomes to be used in interpreting the vascular effects of well‐known or new substances, both in physiological conditions and in a model of homocysteine‐induced pre‐eclampsia.

## 2 MATERIALS AND METHODS

### 2.1 Ethical approval

Umbilical cords were obtained from healthy pregnant women immediately after elective Caesarean section performed in the Clinic for Gynaecology and Obstetrics, University Clinical Centre of the Republic of Srpska (Banja Luka, Bosnia and Herzegovina). Written consent was obtained from all women before the use of their umbilical tissue. Only healthy women who had a Cesarean section between 37 and 39 weeks of gestation were included in the study. The largest number of umbilical samples were segments 10–15 cm long, cut between the placenta and the point of cord transection, while a smaller number of samples were parts closer to the placenta (placental part) or parts closer to the transection point (fetal part). The study was performed in accordance with the standards set by the latest revision of the *Declaration of Helsinki* (except for registration in a database) and was approved by the Ethics Committee of the University Clinical Centre of the Republic of Srpska, Banja Luka (no. 01‐19‐515‐2/20).

### 2.2 Tissue preparation

The samples were instantly transported to Centre for Biomedical Research (Faculty of Medicine, University of Banja Luka, Bosnia and Herzegovina) in dark glass bottles filled with cooled, modified Krebs‐bicarbonate solution. The segments were stored at 0–4°C and used within 24 h after collection.

The HUAs were cleaned of Warthon's jelly and connective tissue and cut into rings (3–4 mm in length). The HUA rings were suspended between two wire hooks in organ bath chambers filled with 10 mL of Krebs‐bicarbonate solution, aerated with a mixture of 95% oxygen and 5% carbon dioxide. One of the hooks was attached to a transducer (MDE, SN: 20K0910) connected to an amplifier (MDE, SN: 20K0912) with a recording system (IsoSys, MDE Research, Budapest, Hungary). Another wire hook was attached to a displacement unit allowing gentle adjustment of passive tension. The optimization of the conditions for measuring the isometric contraction of isolated HUA rings was described earlier (Đukanović et al., 2020).

### 2.3 Solutions for tissue bath system

Krebs‐bicarbonate solution was used for HUA ring nutrition and adaptation during the experiment. The solution was prepared by dissolving chemicals in distilled water to achieve final concentrations as follows (mM): 118.3 NaCl, 4.7 KCl, 1.2 KH_2_PO_4_, 1.2 MgSO_4_, 25 NaHCO_3_, 2.5 CaCl_2_ and 11 glucose. The pH was adjusted to 7.4 with 1 M NaOH and heated to 37°C before use.

A modified Krebs‐bicarbonate solution was used for transport, storage and preparation of isolated HUA tissue. In comparison to the standard Krebs‐bicarbonate solution, the modified solution contained a lower concentration of calcium ions (0.16 mM). It was established previously (Đukanović et al., 2020) that higher concentrations of calcium can cause cell damage and thus endanger the viability of the preparation.

### 2.4 Experimental protocols

The experimental protocols presented in this study were developed for measuring some of the pathophysiological phenomena in the vascular responses of isolated HUA preparations. Protocols were conducted to describe potential outcomes to be used in interpreting the vascular effects of various drugs in physiological conditions and in a homocysteine‐induced model of pre‐eclampsia.

Before the start of the experimental protocols, each HUA ring was prepared according to experimental conditions previously defined in our laboratory (Đukanović et al., 2020). Preparations were allowed to adapt for 30 min, after which they were gradually stretched to the passive tension of 2 g and incubated for 120 min in total. During the incubation period, 40 mmol/L KCl was added at three time points (30, 90 and 120 min). Between each KCl addition, the medium was renewed (every 10 min), and preparations were adjusted once again to 2 g of passive tension.

Several different vasoconstrictors were chosen for the vasoconstriction tests (serotonin, prostaglandin F and phenylephrine), in order to determine and compare their potencies and efficacies. Additionally, the influence of different incubation times on the reactivity of the HUA preparation to serotonin and prostaglanin F was examined.

The relaxing potential of HUA preparations was assessed using the endothelium‐dependent vasodilator acetylcholine and the endothelium‐independent vasodilator minoxidil. To demonstrate the mechanism of minoxidil‐induced vasodilatation in HUAs, well‐known potassium channel blockers were used: glibenclamide (ATP‐sensitive K^+^ channel blocker), tetraethylammonium (BK_Ca_ and K_V_ blocker) and 4‐aminopyridine (4‐AP; K_V_ blocker).

To investigate the role of L‐type voltage‐operated calcium channels in HUA vasoreactivity, experiments were performed using nicardipine and BAY K 8644, a selective blocker and an opener of L‐type Ca^2+^ channels, respectively. An additional set of experiments was performed in Ca^2+^‐free Krebs bicarbonate medium using BaCl_2_ solution.

In order to estimate the impact of homocysteine on serotonin contraction, media with two different concentrations of homocysteine were used for incubation of HUA preparations for 2 h. These short‐term exposures of HUA rings to higher concentrations of homocysteine were also studied on minoxidil‐induced relaxation.

The efficacy of vasoconstrictors was expressed as a percentage relative to the reference KCl‐induced contraction (60 mM KCl), and the relaxation responses were expressed as a percentage of the serotonin precontraction (1 μM). The obtained concentration–response curves were compared with the respective control curves.

### 2.5 Histological examination

After isolation from umbilical cord, HUAs were fixated in 4% formaldehyde for 48 h. Tissue processing was performed in a Laica TP 1020 tissue processor, after which tissue samples were embedded in paraffin blocks and cut into 4‐μm‐thick sections using a Rotary 3003 microtome. All sections were stained with Haematoxylin and Eosin. The analysis of the obtained samples was performed with a Leica DM 6000B binocular microscope, equipped with a Leica DFC310FX camera.

### 2.6 Drugs and solutions

Serotonin (purity ≥98%), prostaglandin F (purity ≥99%), phenylephrine hydrochloride (purity ≥99%), l‐homocysteine (purity ≥98%), nicardipine (purity ≥98%), barium chloride (purity ≥99.9%), tetraethylammonium (TEA; purity ≥99.5%), 4‐aminopyridine (4‐AP; purity ≥99%) and glibenclamide (purity ≥ 99) were purchased from Sigma Aldrich (St. Louis, MO, USA). Minoxidil (purity ≥99%) was generously donated from the pharmaceutical company Bosnalijek (Sarajevo, Bosnia and Herzegovina), and (±)BAY K 8644 (purity ≥97.5%) was purchased from Cayman Chemical Company. Their stock solutions were prepared either in distilled water or in 99.9% ethanol (with the exception of minoxidil, for which a stock solution was prepared in propylene glycol), according to their solubility, and the subsequent dilutions were carried out in distilled water. The final concentrations of solvent never exceeded 0.33% in the 10 mL organ bath. All solutions were kept at 0–4°C when not used in experiments.

### 2.7 Statistics

Statistical analysis and graphs were prepared using the software SigmaPlot v.11 (Systat Software). Results were summarized as the mean (SD) of *n* replicates, where the *n* is the number of HUA rings tested in one protocol, each obtained from a different umbilical cord. Statistical comparisons were performed using Student's paired *t*‐test or two‐way ANOVA, followed by Bonferroni test, and differences were considered statistically significant at *P*‐values of <0.05.

## 3 RESULTS

### 3.1 Vascular response of HUA to typical vasoconstrictors

**FIGURE 1:**
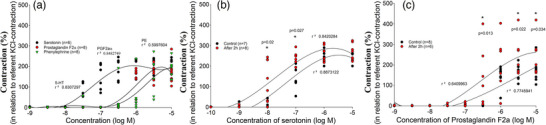
Vascular response of human umbilical artery (HUA) to typical vasoconstrictors. (a) Concentration–contraction curves for serotonin (*n =* 6), prostaglandin F (*n =* 8) and phenylephrine (*n =* 8) obtained in HUA rings. (b, c) Concentration–response curves obtained when the vasoconstrictor was added to the system in the regular manner (control) and when it was added after an additional 2 h of incubation, for serotonin (b) and prostaglandin F (c). Responses are expressed as the percentage of reference contraction (100%) induced by 6 × 10^−2^ M KCl; *n* is the number of HUA preparations studied, each obtained from a different umbilical cord, and the coefficient of determination (*r* ^2^) and *P*‐values are indicated numerically where applicable.

Different concentration‐dependent vasoconstriction responses were obtained for serotonin, prostaglandin F and phenylephrine (Figure 1a). Serotonin exhibited the highest potency, and all vasoconstrictors had similar maximum efficacy, which was ∼200% higher than KCl‐induced vasoconstriction (Table 1).

**TABLE 1:** Reactivity of human umbilical artery preparations to typical vasoconstrictors and vasodilators.

*Tested compound*	*Mean (%)*	*SD (%)*	*n*	*Response*
*Serotonin*	223.83	70.69	7	*Contraction*
*Prostaglandin F*	152.86	61.04	8	
*Phenylephrine* ^a^	191.24	104.16	9	
*Phenylephrine* ^b^	19.79	15.58	9	
*Nicardipine + serotonin*	135.67	61.11	5	
*Homocysteine (100 μM) + serotonin*	286.54	90.54	10	
*Homocysteine (300 μM) + serotonin*	224.59	68.35	7	
*Acetylcholine* ^a^	85.86	4.16	4	*Relaxation*
*Acetylcholine* ^b^	4.98	3.41	14	
*Minoxidil*	81.78	37.69	19	
*Glibenclamide + minoxidil*	50.39	12.03	7	
*4‐AP + minoxidil*	38.82	24.04	6	
*TEA + minoxidil*	78.59	50.52	6	
*Homocysteine + minoxidil*	29.46	10.55	6	

*Note*: The efficacy of selected vasoconstrictors was expressed in relationship to the reference KCl‐induced contraction (100%), and the efficacy of vasodilators was expressed as a decrease in serotonin‐induced precontraction (100%); *n* is the number of HUA preparations, each obtained from a different umbilical cord.

^a^ Responsive preparations.

^b^ Non‐responsive preparations.

**FIGURE 2:**
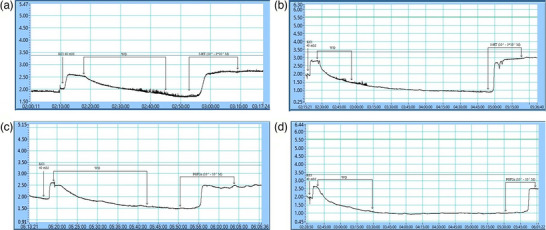
Representative data traces recorded by IsoSys, MDE Research software. The data traces show significant potentiation of the activity and efficiency of vasoconstrictors in the human umbilical artery preparations that were incubated for an additional period. (a) Serotonin was added immediately after the regular stabilization period. (b) Serotonin was added after an additional 2 h stabilization period. (c) Prostaglandin F was added immediately after the regular stabilization period. (d) Prostaglandin F was added after an additional 2 h stabilization period.

Significant potentiation of the activity and efficiency of both serotonin (*P* = 0.027) and prostaglandin F (*P* = 0.034) was observed in the preparations that were incubated for an additional 2 h compared with the controls (Figure 2). Additional incubation of HUA preparations resulted in left‐shifting of the concentration–response curves for serotonin and prostaglandin F, compared with the control curves (Figure 1b, c).

**FIGURE 3:**
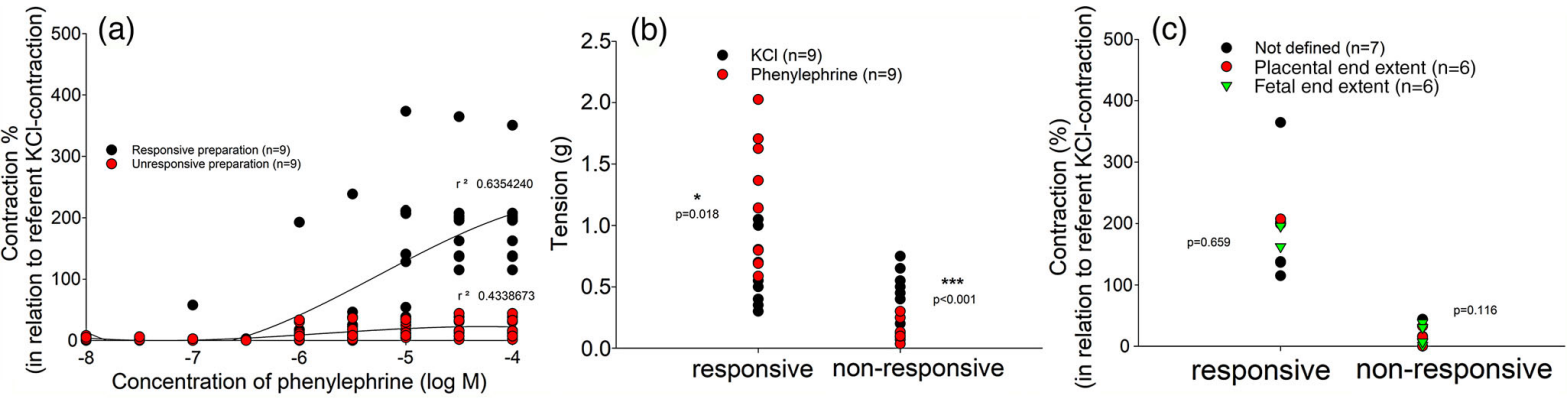
Human umbilical artery (HUA) variability in the responses to phenylephrine. (a) Concentration–contraction curves for phenylephrine (responsive preparation, *n =* 9, and non‐responsive preparation, *n =* 9) obtained in HUA rings. (b) Vascular responses of responsive and unresponsive preparations of HUAs in the presence of 6 × 10^−2^ M KCl and 10^−5^ M phenylephrine. (c) Vascular responses of non‐defined HUA preparations, and preparations of the fetal end or the placental end in the presence of 10^−5^ M phenylephrine. Contractions are expressed as a percentage of the reference contraction (100%) induced by 6 × 10^−2^ M KCl (a, c) or in grams of tension (b); *n* is the number of HUA preparations studied, each obtained from a different umbilical cord, and the coefficient of determination (*r* ^2^) and *P*‐values are indicated numerically where applicable.

It was found that in half of the HUA preparations examined, phenylephrine caused significant vasoconstriction (∼200% of maximal KCl‐induced contraction), whereas in the other half of the HUA preparations, there was no significant response to phenylephrine at all (Figure 3a). However, all preparations unresponsive to phenylephrine exhibited significant contractions in the presence of hypertonic potassium (*P* < 0.001), thus confirming their ability to activate the contractile apparatus (Figure 3b). Owing to the variability of the responses, it was assumed that different parts of the umbilical cord show different vascular reactivity to the presence of phenylephrine. Therefore, HUA preparations from the placental or fetal part were selected and their reactivity to phenylephrine was compared. However, no statistical significance (*P* = 0.659) was found between different parts of HUA in responses to phenylephrine (Figure 3c).

### 3.2 Vascular response of the HUA to vasodilators: Acetylcholine and minoxidil

**FIGURE 4:**
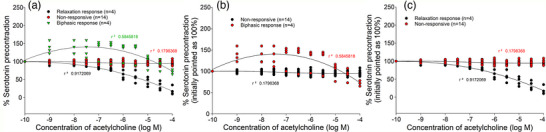
Concentration–response curves for acetylcholine applied to human umbilical artery (HUA) rings with different vascular reactivity. (a) Comparative presentation of non‐responsive rings (*n =* 14) and rings with biphasic (*n =* 4) and relaxation (*n =* 4) responses. (b) Comparative demonstration of concentration–response curves for non‐responsive rings (*n =* 14) and HUA rings with a biphasic response (contraction preceding relaxation; *n =* 4). (c) HUA rings with a strong relaxation response. Responses are expressed as a decrease in serotonin precontraction (100%) induced by 10^−6^ M serotonin; *n* is the number of HUA preparations studied, each obtained from a different umbilical cord, and the coefficient of determination (*r* ^2^) is indicated numerically for each concentration–response curve.

The differential vascular reactivity of >20 HUA rings, each from a different donor, tested in the presence of acetylcholine, was confirmed. The largest number of HUA rings showed weak relaxation (<10%) or there was a complete lack of response to acetylcholine, and such preparations were classified as non‐responsive (Table 1). However, a smaller number of preparations showed a pronounced reactivity, some with significant relaxation and others with biphasic activity (Figure 4a).

Preparations with biphasic responses at low concentrations of acetylcholine (10^−9^ M) showed contractions that were ∼50% of the serotonin‐induced precontraction. These contraction responses were sustained to a concentration of acetylcholine of 30 μM, after which a relaxation occurred. The final relaxation response at the highest concentration of acetylcholine (10^−4^ M) reached >70% of the serotonin‐induced precontraction (Figure 4b). Concentration‐dependent relaxations were registered in HUA rings with a typical relaxation response, where at the highest concentration of acetylcholine, >80% decrease in serotonin‐induced precontraction was achieved (Figure 4c).

**FIGURE 5:**
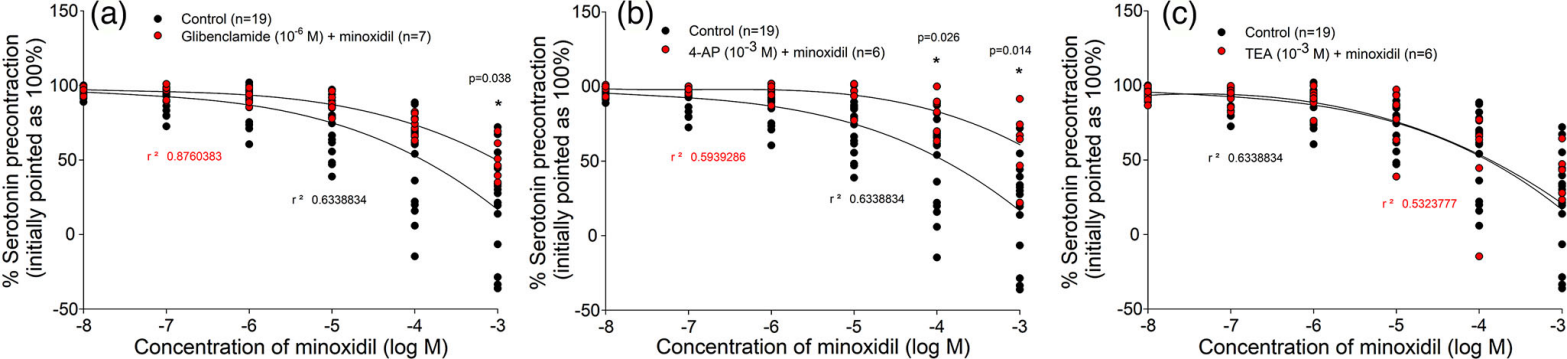
Concentration–response curves obtained in human umbilical artery (HUA) rings to minoxidil in the absence (*n =* 19) and presence of: (a) glibenclamide (*n =* 7); (b) 4‐aminopyridine (4‐AP; *n =* 6); and (c) TEA (*n =* 6). Responses are expressed as a decrease in serotonin precontraction (100%) induced by 10^−6^ M serotonin; *n* is the number of HUA preparations studied, each obtained from a different umbilical cord, and the coefficient of determination (*r* ^2^) and *P*‐values are indicated numerically where applicable.

The control concentration–response curve for minoxidil showed that at concentration of 10^−4^ M, minoxidil caused a 50% relaxation, and at an extremely high concentration (10^−3^ M) it caused almost 100% relaxation. The possible role of K_ATP_, K_V_ and/or BK_Ca_ channels in the mechanism of minoxidil‐induced relaxation of HUA was investigated by using specific blockers, such as glibenclamide, 4‐AP and TEA, respectively. The incubation of HUA rings with glibenclamide (10^−6^ M) resulted in a less pronounced relaxation in response to minoxidil, which was significant, in comparison to the control group at a concentration of 10^−3^ M (*P* = 0.038; Figure 5a). Likewise, incubation of HUA rings with the K_V_ channel blocker 4‐AP (10^−3^ M) showed significant differences in the minoxidil‐induced relaxation at concentrations of 10^−4^ M (*P* = 0.026) and 10^−3^ M (*P* = 0.014; Figure 5b). However, the blocking of BK_Ca_ channels by 10^−3^ M TEA had no effect on the relaxation obtained with minoxidil (Figure 4c).

### 3.3 Role of voltage‐dependent Ca^2+^ channels in the HUA contraction

**FIGURE 6:**
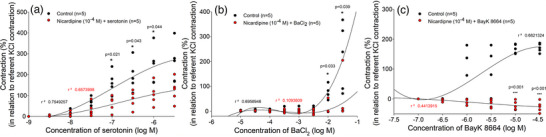
Role of voltage‐dependent Ca^2+^ channels in contraction of the human umbilical artery (HUA). Concentration–contraction curves were obtained in HUA rings for: (a) serotonin in the absence (*n =* 5) and presence of nicardipine (*n =* 5); (b) BaCl_2_ in the absence (*n =* 5) and presence of nicardipine (*n =* 5); and (c) BAY K 8864 in the absence (*n =* 5) and presence of nicardipine (*n =* 5). Responses are expressed as a percentage of the reference contraction (100%) induced by 6 × 10^−2^ M KCl; *n* is the number of HUA preparations studied, each obtained from a different umbilical cord, and coefficient of determination (*r* ^2^) and *P*‐values are indicated numerically where applicable.

The role of L‐type Ca^2+^ channels in the smooth muscle contraction of HUAs was investigated. The incubation of HUA rings with nicardipine (10^−4^ M) resulted in decreased serotonin contraction, which was significant at concentrations of 10^−7^ M (*P* = 0.021), 3 × 10^−6^ M (*P* = 0.043) and 10^−6^ M (*P* = 0.044; Figure 6a). Additionally, HUA preparations were contracted by using the L‐type Ca^2+^ channel agonist BaCl_2_ or the L‐type Ca^2+^ channel opener BAY K 8864. By comparing their control concentration–response curves with the curves in the presence of nicardipine, a strong suppression of contraction of the HUA rings in the presence of L‐type Ca^2+^ channel blocker was confirmed (Figure 6b, c). Interestingly, nicardipine (10^−4^ M) completely inhibited the contractions promoted by BAY K 8864 at concentrations of 10^−5^ M (*P* < 0.001) and 3 × 10^−5^ M (*P* < 0.001), whereas the suppressive effect was slightly weaker in the BaCl_2_‐induced contraction (at a BaCl_2_ concentration of 3 × 10^−2^ M, *P* = 0.039), suggesting that, in addition to L‐type Ca^2+^ channels, some other channels also contribute to its contractile properties.

### 3.4 Vascular responses of HUA in presence of homocysteine

**FIGURE 7:**
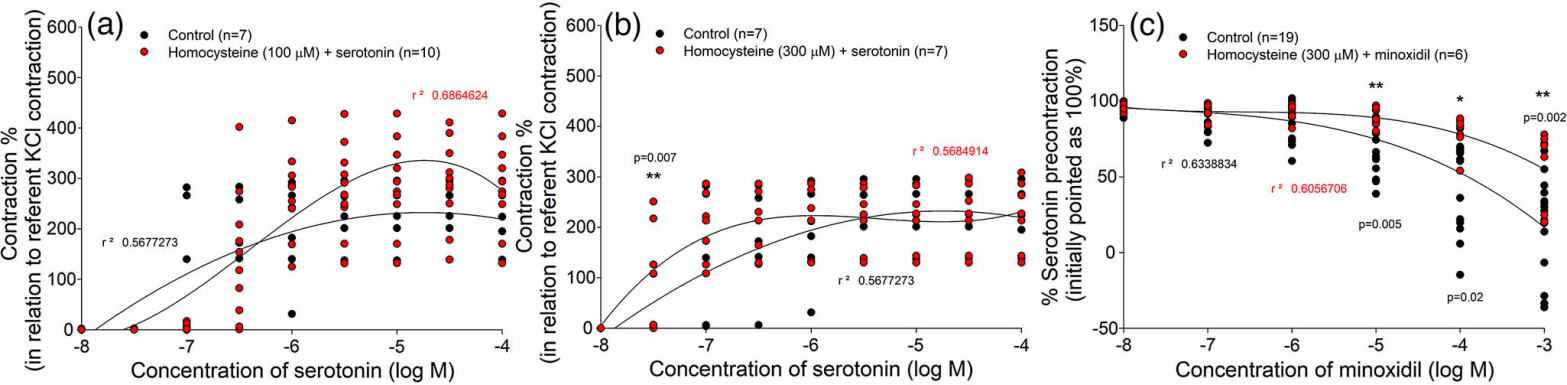
Vascular response of human umbilical artery (HUA) in the presence of homocysteine. Concentration–contraction curves were obtained in HUA rings for serotonin in the absence (*n =* 7) and presence of homocysteine: (a) at concentration of 100 μM (*n =* 10); and (b) at concentration of 300 μM (*n =* 7). (c) Cumulative log concentration–response curves of minoxidil in the absence (*n =* 19) and presence of homocysteine (300 μM) (*n =* 6). Responses are expressed as a percentage of the reference contraction (100%) induced by 6 × 10^−2^ M KCl (a, b) or as a decrease in serotonin precontraction (100%) induced by 10^−6^ M serotonin (c); *n* is the number of HUA preparations studied, each obtained from a different umbilical cord, and coefficient of determination (*r* ^2^) and *P*‐values are indicated numerically where applicable.

Homocysteine in a concentration of 100 μM did not show a significant effect on serotonin‐induced contraction (*P* = 0.195; Figure 7a). However, when applied at a concentration of 300 μM, homocysteine caused a significant leftward shift of the serotonin concentration–response curve (*P* = 0.007; Figure 7b). However, short‐term (2 h) incubation of HUA rings with homocysteine (300 μM) resulted in less vascular relaxation in response to minoxidil, which was significant in comparison to the control group at minoxidil concentrations of 10^−5^ M (*P* = 0.005), 10^−4^ M (*P* = 0.02) and 10^−3^ M (*P* = 0.002; Figure 7c).

### 3.5 Histological analysis

**FIGURE 8:**
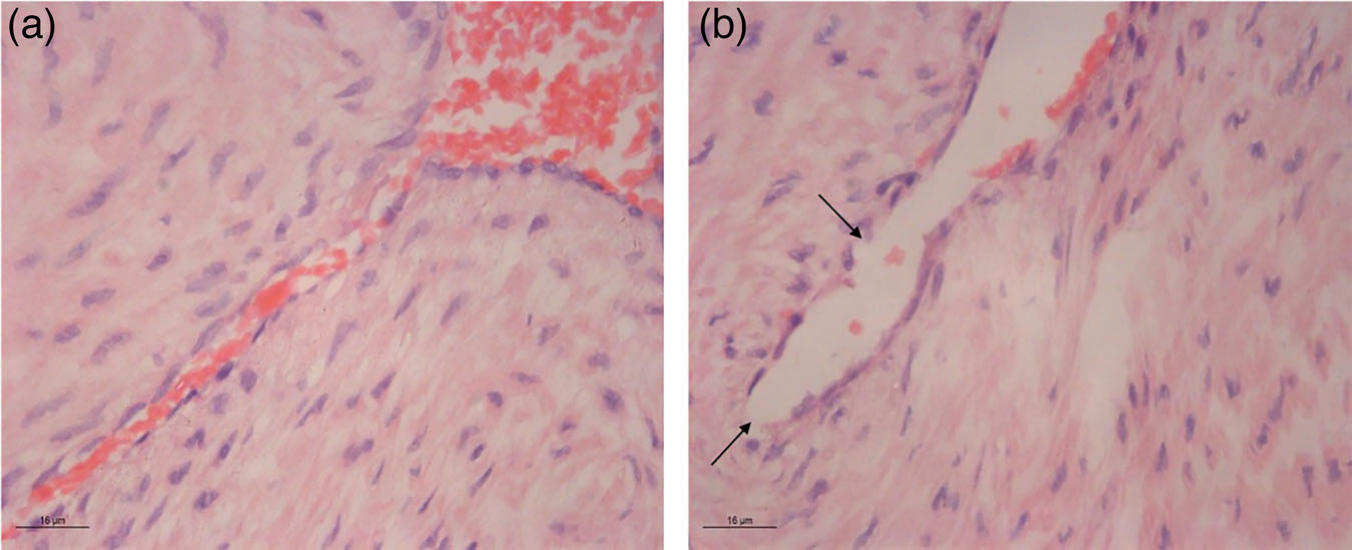
Histological analysis for human umbilical artery cross‐sections. (a) Normal histological findings for the inner layer. (b) Shattered endothelial layer (arrows). Haematoxylin and Eosin staining, ×63 magnification.

The layers of the arterial wall with a narrowed lumen, a typical irregular branched shape filled with numerous erythrocytes, can be observed clearly in the cross‐section of the HUA (Figure 8). The inner layer is composed of endothelial cells with a prominent nucleus and sparse cytoplasm, lying on a band of subendothelial connective tissue of variable thickness (Figure 8a). A well‐developed media is composed of longitudinal bundles of poorly differentiated smooth muscle cells and spirally coiled smooth muscle cells. The only difference that could be noticed between individual tissue samples was in the inner layer of the HUA, where a discontinuity of the endothelial layer was observed (Figure 8b).

## 4 DISSCUSION

It is generally known that serotonin is the strongest vasoconstrictor of HUAs and that 5‐HT_2A_ and, to some extent, 5‐HT_1B/D_ receptors are responsible for its vasoactivity in HUAs (Gupta et al., 2006; Santos‐Silva et al., 2009). The first set of results of this study confirmed that serotonin induced the most pronounced vasoconstriction of HUAs, with a greater potency and efficacy than prostaglandin F and phenylephrine (Hehir et al., 2009). In addition, it was observed that the vascular responses of HUA preparations were significantly more pronounced when the preparations were also stabilized in Krebs‐bicarbonate solution. Multiple washing, combined with multiple ‘challenging’ with KCl, resulted in serotonin‐induced contractions being more potent and prostaglandin F‐induced contractions more efficient, in comparison to controls (Medina Leyte et al., 2020).

Although the umbilical cord is not innervated, numerous studies indicate that neurotransmitters, such as acetylcholine and catecholamines, can cause contractile responses in human umbilical arteries and veins (Arun et al., 2019; Errasti et al., 2003; Pujol Lereis et al., 2006; Yoshikawa & Chiba, 1991). Considering that only high concentrations of these substances are able to produce contractile effects in vitro, their physiological role is thought to be probably negligible or minimal (Altura et al., 1972).

The phenomenon of variability in the HUA response to phenylephrine, previously described by Altura et al. (1972), was confirmed in our protocol. It was found that approximately half of the preparations reacted to phenylephrine, depending on the concentration, and that the other half of the preparations showed no response. It was established previously that in tissue bath conditions of a higher partial pressure of oxygen (∼120 mmHg), adrenaline caused significant contractions of HUA rings, whereas at a lower partial pressure of oxygen (∼20 mmHg) there was no response to the presence of adrenaline (McGrath et al., 1988). In our study, we could not determine a connection between the part of the umbilical cord used (fetal or placental end), in terms of different receptor expression. It is possible that the differences in the constant oxygen pressure between the experimental sets could explain the variability of HUA responses to phenylephrine.

Another example of HUA response variability was found for acetylcholine. Different results of HUA vasoactivity to the presence of increasing concentrations of acetylcholine have been reported, such as an absence of response to acetylcholine, relaxation with low sensitivity followed by contraction, or incomplete relaxation (Gokhale et al., 1966; Xie & Triggle, 1994). The conduction of our protocol confirmed the variability of responses in the HUA preparations, whereby the largest number of HUA rings did not respond to the presence of acetylcholine. A smaller number of preparations showed pronounced reactivity, some with significant dose‐dependent relaxation, whereas others registered significant contractions followed by relaxation. However, there are findings that clearly indicate that acetylcholine causes only contractions in the HUA rings mediated by activation of muscarinic M_1_ and M_3_ receptors, which are endothelium independent and antagonized by atropine (Chen et al., 2015; Pujol Lereis et al., 2006; Yoshikawa & Chiba, 1991). Variability in acetylcholine responses is probably conditioned by structural and morphological differences between individual HUA segments, considering that the relaxation and contraction effects depend on whether muscarinic receptors on the endothelium or smooth muscle cells are stimulated. Histological analyses of HUA sections revealed differences in endothelial integrity.

Being dominant in HUA smooth muscle cell membranes, the K^+^ channels represent one of the main mechanisms of vascular tone regulation in physiological and pathophysiological conditions (Lorigo et al., 2020; Martín et al., 2014; Saldanha et al., 2013). Therefore, the demonstration of their role in this experimental protocol is based on the vascular effects of minoxidil, a direct arterial vasodilator that effectively opens K^+^ channels modulated by ATP, thus allowing K^+^ efflux and smooth muscle relaxation (McComb et al., 2016). Using selective blockers, such as glibenclamide, 4‐AP and TEA, it was shown that in addition to the expected role of K_ATP_ channels, the voltage‐dependent K_V_ channels also participate in the minoxidil‐induced relaxation, whereas BK_Ca_ channels have no participation in the mechanism of minoxidil‐induced relaxation in HUAs.

Incubation of HUA rings with the selective calcium blocker nicardipine caused a significant suppression of serotonin‐induced contraction, indicating the importance of L‐type Ca^2+^ channels for the contractions mediated by 5‐HT_2A_ and 5‐HT_1B/D_ receptor activation. It was confirmed that, in addition to the intracellular release of Ca^2+^, through the activation of phospholipase C, which also leads to the breakdown of inositol phospholipids and synthesis of inositol 1,4,5,‐trisphosphate, the extracellular Ca^2+^ also plays an important role in the serotonin‐induced contraction of HUAs (Berridge, 1993; Doğan et al., 1991). Experimental protocols performed in Ca^2+^‐free Krebs‐bicarbonate solution are interesting, providing insight into the role of extracellular and/or intracellular Ca^2+^ in the contractile activity of the tested compound. In this protocol, we showed that nicardipine not only suppresses serotonin contraction, but also significantly suppresses BaCl_2_‐induced contraction and completely blocks the contraction induced by the L‐type Ca^2+^ channel agonist BAY K 8644, indicating that the contractile mechanisms in HUAs are similar to those in other vascular smooth muscle cells (Tiritilli, 1998).

Hyperhomocysteinaemia has long been recognized as an important and independent risk factor for vascular diseases, and oxidative stress has been identified as a possible mechanism for homocysteine‐induced endothelial dysfunction (Okatani et al., 2000). The direct contractile effects of homocysteine on isolated HUAs have been described previously and refer to a concentration‐dependent potentiation of prostaglandin F‐induced contraction, in addition to a significant reduction of the relaxation induced by the Ca^2+^ ionophore A23187 (Okatani et al., 2000). In the protocols presented here, we confirmed the procontractile effect of homocysteine on HUA preparations, showing that in its presence, serotonin‐induced contractions were significantly potentiated and minoxidil‐induced relaxations were significantly reduced. Homocysteine, at 100 μM, enhanced the contractile response of isolated human internal mammary artery to U46619, a thromboxane A2 mimetic, and impaired the acetylcholine‐induced relaxation, which is associated with loss of the BK_Ca_ β1‐subunit (Sun et al., 2021). However, in our set of experiments, in order to achieve significance in the vascular effects of homocysteine, it was necessary to apply a threefold higher concentration of homocysteine (300 μM), which was determined by using different concentrations and incubation periods, as confirmed by Radenković et al. (2014).

## 5 CONCLUSION

The proper incubation conditions (e.g., temperature, oxygen levels), followed by a KCl potentiation pattern of HUA contractions, are proposed as cornerstone steps in obtaining optimal experimental results. Additionally, differences in endothelial integrity have to be considered if investigating endothelium‐dependent vasodilatation. In this case, pathohistological analysis of HUA preparations is advised.

Undoubtedly, the need for in vitro studies focused on discovering new vasodilator compounds is growing, and HUA preparations as a model have multiple advantages. The presented experimental protocols reveal doubts about some of the methodological challenges in working with HUAs. The obtained results could be useful for planning future in vitro studies, in terms of both investigating the molecules with vasodilatory properties and testing different compounds with the potential to induce vasoconstriction.

## AUTHOR CONTRIBUTIONS

All experiments were performed in the Centre for Biomedical Research, Faculty of Medicine, University of Banja Luka, The Republic of Srpska, Bosnia and Herzegovina. Each author's contribution to the paper is quantified as follows: M.G.B.: Methodology, Data curation, Formal analysis, Investigation, Writing—original draft; Đ.Đ., S.M., S.J.: Formal analysis, Investigation, Writing—original draft; D.M.Dj., M.P.S. and R.Š.: Conceptualisation, Methodology, Supervision, Writing—review & editing). All authors approved the final version of the manuscript and agree to be responsible for all aspects of the work in ensuring that questions related to the accuracy or integrity of any part of the work are appropriately investigated and resolved. All persons designated as authors qualify for authorship, and all those who qualify for authorship are listed.

## CONFLICT OF INTEREST

The authors declare no conflicts of interest.

## FUNDING INFORMATION

None.

## Data Availability

All original “raw” data supporting the results presented in this study are fully available to The Journal upon reasonable request.

## References

[eph13435-bib-0001] Altura, B. M. , Malaviya, D. , Reich, C. F. , & Orkin, L. R. (1972). Effects of vasoactive agents on isolated human umbilical arteries and veins. The American Journal of Physiology, 222(2), 345–355. 4400488 10.1152/ajplegacy.1972.222.2.345

[eph13435-bib-0002] Arun, O. , Taylan, S. , Duman, I. , Oc, B. , Yilmaz, S. , Tekin, A. , Celik, C. , Bariskaner, H. , & JB1, C. (2019). In vitro vasoactive effects of dexmedetomidine on isolated human umbilical arteries. Bratislavske lekarske listy, 120(1), 40–45. 30685991 10.4149/BLL_2019_006

[eph13435-bib-0003] Berridge, M. J. (1993). Inositol trisphosphate and calcium signalling. Nature, 361(6410), 315–325. 8381210 10.1038/361315a0

[eph13435-bib-0004] Bertrand, C. , Duperron, L. , & St‐Louis, J. (1993). Umbilical and placental vessels: Modifications of their mechanical properties in preeclampsia. American Journal of Obstetrics and Gynecology, 168(5), 1537–1546. 8498440 10.1016/s0002-9378(11)90795-9

[eph13435-bib-0005] Chen, N. , Lv, J. , Bo, L. , Li, N. , Wu, C. , Yin, X. , Li, J. , Tao, J. , Chen, J. , He, Y. , Huang, S. , Xiao, J. , Mao, C. , & Xu, Z. (2015). Muscarinic‐mediated vasoconstriction in human, rat and sheep umbilical cords and related vasoconstriction mechanisms. BJOG: An International Journal of Obstetrics and Gynaecology, 122(12), 1630–1639. 25403992 10.1111/1471-0528.13144

[eph13435-bib-0006] Doğan, N. , Ciçek, E. , Cenik, A. G. , Singirik, E. , Kiliç, M. , & Ozcan, A. S. (1991). No Title5‐Hydroxytryptamine‐induced contraction of human isolated umbilical artery and its dependence on cellular and extracellular Ca++. Archives Internationales De Pharmacodynamie Et De Therapie, 312, 79–85. 1772342

[eph13435-bib-0007] Đukanović, Đ. , Bojić, M. G. , Marinković, S. , Trailović, S. , Stojiljković, M. P. , & Škrbić, R. (2022). Vasorelaxant effect of monoterpene carvacrol on isolated human umbilical artery. Canadian Journal of Physiology and Pharmacology, 100(8), 755–762. 35507953 10.1139/cjpp-2021-0736

[eph13435-bib-0008] Đukanović, Đ. , Gajić, M. , & Škrbić, R. (2020). Time‐dependent and force‐dependent vasoreactivity of isolated human umbilical arteries. Scripta Medica, 51(3), 134–140.

[eph13435-bib-0009] Errasti, A. E. , Werneck De Avellar, M. C. , Daray, F. M. , Tramontano, J. , Luciani, L. I. , Bard Lina, M. J. , Maróstica, E. , & Rothlin, R. P. (2003). Human umbilical vein vasoconstriction induced by epinephrine acting on α1B‐adrenoceptor subtype. American Journal of Obstetrics and Gynecology, 189(5), 1472–1480. 14634588 10.1067/s0002-9378(03)00646-x

[eph13435-bib-0010] Evaristo, R. , Silva, A. D. A. , Pereira‐de‐morais, L. , Almeida, N. D. S. , Iriti, M. , Kerntopf, M. R. , Rose, I. , Menezes, A. D. , Douglas, H. , Coutinho, M. , & Barbosa, R. (2020). Relaxant Effect of Monoterpene (−)‐Carveol on.pdf. Molecules (Basel, Switzerland), 25(2682), 1–11. 10.3390/molecules25112681 PMC7321233 32527034

[eph13435-bib-0011] Gokhale, S. D. , Gulati, O. D. , Kelkar, L. V. , & Kelkar, V. V. (1966). Effect of some drugs on human umbilical artery in vitro. British Journal of Pharmacology and Chemotherapy, 27(2), 332–346. 19108217 10.1111/j.1476-5381.1966.tb01666.x PMC1510819

[eph13435-bib-0012] Gupta, S. , Hanff, L. M. , Visser, W. , Steegers, E. A. P. , Saxena, P. R. , Vulto, A. G. , & Maassenvandenbrink, A. (2006). Functional reactivity of 5‐HT receptors in human umbilical cord and maternal subcutaneous fat arteries after normotensive or pre‐eclamptic pregnancy. Journal of Hypertension, 24(7), 1345–1353. 16794484 10.1097/01.hjh.0000234115.40648.88

[eph13435-bib-0013] Hehir, M. P. , Moynihan, A. T. , Glavey, S. V. , & Morrison, J. J. (2009). Umbilical artery tone in maternal obesity. Reproductive Biology and Endocrinology, 7, 1–7. 19161625 10.1186/1477-7827-7-6 PMC2649927

[eph13435-bib-0014] Jin, L. , Lipinski, A. , & Conklin, D. (2018). A simple method for normalization of aortic contractility. Journal of Vascular Research, 55(3), 177–186. 29975955 10.1159/000490245 PMC6054883

[eph13435-bib-0015] Lorigo, M. , Oliveira, N. , & Cairrao, E. (2020). Clinical importance of the human umbilical artery potassium channels. Cells, 9(9), 4–8. 10.3390/cells9091956 PMC7565333 32854241

[eph13435-bib-0016] Martín, P. , Rebolledo, A. , Palomo, A. R. R. , Moncada, M. , Piccinini, L. , & Milesi, V. (2014). Diversity of potassium channels in human umbilical artery smooth muscle cells: A review of their roles in human umbilical artery contraction. Reproductive Sciences, 21(4), 432–441. 24084522 10.1177/1933719113504468 PMC3960844

[eph13435-bib-0017] McComb, M. N. , Chao, J. Y. , & Ng, T. M. H. (2016). Direct vasodilators and sympatholytic agents. Journal of Cardiovascular Pharmacology and Therapeutics, 21(1), 3–19. 26033778 10.1177/1074248415587969

[eph13435-bib-0018] McGrath, J. C. , MacLennan, S. J. , & Whittle, M. J. (1988). Comparison of the effects of oxygen, 5‐hydroxytryptamine, bradykinin and adrenaline in isolated human umbilical artery smooth muscle. Quarterly Journal of Experimental Physiology, 73(4), 547–559. 3174915 10.1113/expphysiol.1988.sp003175

[eph13435-bib-0019] Medina Leyte, D. J. , Domínguez Pérez, M. , Mercado, I. , Villarrreal Molina, M. T. , & Jacobo Albavera, L. (2020). Use of human umbilical vein endothelial cells (HUVEC) as a model to study cardiovascular disease. Applied Sciences, 10, 938.

[eph13435-bib-0020] Okatani, Y. , Wakatsuki, A. , & Reiter, R. J. (2000). Protective effect of melatonin against homocysteine‐induced vasoconstriction of human umbilical artery. Biochemical and Biophysical Research Communications, 277(2), 470–475. 11032746 10.1006/bbrc.2000.3687

[eph13435-bib-0021] Pereira‐de‐Morais, L. , Silva, A. D. A. , Mikevely de Sena Bastos, C. , Lucena Calixto, G. , Moura Araújo, I. , Cavalcante Araújo, M. , Barbosa, R. , & Leal‐Cardoso, J. H. (2023). The preeclampsia condition alters external potassium‐evoked contraction of human umbilical vessels. Placenta, 138, 68–74. 37209614 10.1016/j.placenta.2023.05.005

[eph13435-bib-0022] Pujol Lereis, V. A. , Hita, F. J. , Gobbi, M. D. , Verdi, M. G. , Rodriguez, M. C. , & Rothlin, R. P. (2006). Pharmacological characterization of muscarinic receptor subtypes mediating vasoconstriction of human umbilical vein. British Journal of Pharmacology, 147(5), 516–523. 16444291 10.1038/sj.bjp.0706654 PMC1616972

[eph13435-bib-0023] Radenković, M. , Djurić, D. , Janković, R. , & Prostran, M. (2014). The analysis of transduction mechanisms associated with an acute action of homocysteine on isolated rat femoral artery. Acta Physiologica Hungarica, 101(4), 448–460. 25532956 10.1556/APhysiol.101.2014.4.6

[eph13435-bib-0024] Saldanha, P. A. , Cairrão, E. , Maia, C. J. , & Verde, I. (2013). Long‐ and short‐term effects of androgens in human umbilical artery smooth muscle. Clinical and Experimental Pharmacology and Physiology, 40(3), 181–189. 23278339 10.1111/1440-1681.12047

[eph13435-bib-0025] Santos‐Silva, A. J. , Cairrão, E. , Marques, B. , & Verde, I. (2009). Regulation of human umbilical artery contractility by different serotonin and histamine receptors. Reproductive Sciences, 16(12), 1175–1185. 19801536 10.1177/1933719109343787

[eph13435-bib-0026] Santos‐Silva, A. J. , Cairrão, E. , Morgado, M. , Álvarez, E. , & Verde, I. (2008). PDE4 and PDE5 regulate cyclic nucleotides relaxing effects in human umbilical arteries. European Journal of Pharmacology, 582(1–3), 102–109. 18234184 10.1016/j.ejphar.2007.12.017

[eph13435-bib-0027] Stefos, T. , Sotiriadis, A. , Vasilios, D. , Tsirkas, P. , Korkontzelos, I. , Avgoustatos, F. , & Lolis, D. (2003). Umbilical cord length and parity—The Greek experience. European Journal of Obstetrics Gynecology and Reproductive Biology, 107(1), 41–44. 12593892 10.1016/s0301-2115(02)00307-x

[eph13435-bib-0028] Sun, W.‐T. , Xue, H.‐M. , Hou, H.‐T. , Chen, H.‐X. , Wang, J. , He, G.‐W. , & Yang, Q. (2021). Homocysteine alters vasoreactivity of human internal mammary artery by affecting the KCa channel family. Annals of Translational Medicine, 9(8), 625–625. 33987323 10.21037/atm-20-6821 PMC8106027

[eph13435-bib-0029] Tiritilli, A. (1998). Effects of magnesium on human umbilical arteries: Role of intracellular calcium stores. Pharmacology, 57(6), 295–304. 9792970 10.1159/000028255

[eph13435-bib-0030] Upchurch, G. R. , Welche, G. N. , Fabian, A. J. , Freedman, J. E. , Johnson, J. L. , Keaney, J. F. , & Loscalzo, J. (1997). Homocyst(e)ine decreases bioavailable nitric oxide by a mechanism involving glutathione peroxidase. Journal of Biological Chemistry, 272(27), 17012–17017. 9202015 10.1074/jbc.272.27.17012

[eph13435-bib-0031] Xie, H. , & Triggle, C. R. (1994). Endothelium‐independent relaxations to acetylcholine and A23187 in the human umbilical artery. Journal of Vascular Research, 31(2), 92–105. 8117864 10.1159/000159035

[eph13435-bib-0032] Yoshikawa, F. , & Chiba, S. (1991). Pharmacological analysis of vasoconstrictor responses of isolated and perfused human umbilical arteries. Heart and Vessels, 6(4), 197–202. 1800478 10.1007/BF02125097

